# Innexins: Expression, Regulation, and Functions

**DOI:** 10.3389/fphys.2018.01414

**Published:** 2018-10-11

**Authors:** Juan Güiza, Iván Barría, Juan C. Sáez, José L. Vega

**Affiliations:** ^1^Laboratorio de Fisiología Experimental, Instituto Antofagasta, Universidad de Antofagasta, Antofagasta, Chile; ^2^Departamento de Fisiología, Pontificia Universidad Católica de Chile, Santiago, Chile; ^3^Instituto de Neurociencias, Centro Interdisciplinario de Neurociencias de Valparaíso, Universidad de Valparaíso, Valparaíso, Chile

**Keywords:** gap junction, pannexin, connexin, invertebrates, channels, hemichannels

## Abstract

The innexin (Inx) proteins form gap junction channels and non-junctional channels (named hemichannels) in invertebrates. These channels participate in cellular communication playing a relevant role in several physiological processes. Pioneer studies conducted mainly in worms and flies have shown that innexins participate in embryo development and behavior. However, recent studies have elucidated new functions of innexins in *Arthropoda, Nematoda, Annelida*, and *Cnidaria*, such as immune response, and apoptosis. This review describes emerging data of possible new roles of innexins and summarizes the data available to date.

## Introduction

Intercellular communication plays a key role in many cell functions (Dykes and Macagno, 2006). Innexins are integral membrane proteins that participate in cellular communication, forming gap junction channels, and hemichannels (Vega et al., 2013). Gap junction channels allow the diffusion of ions, second messengers and small molecules between adjacent cells (Sáez et al., 2003), whereas hemichannels (also named innexons) allow the exchange between the cell interior with the extracellular milieu (Dahl and Muller, 2014). Krishnan and collaborators described the first innexin in 1993. They studied a mutant fly that failed to respond to a light-off stimulus and identified a gene called *Pas* (for Passover, also known as shaking-B), which encodes a protein of 361 amino acids and is expressed in giant fibers. Notably, this mutation causes synaptic dysfunction in giant fiber neurons, which are responsible for coordinating the response promoted by light-off (Krishnan et al., 1993). Subsequently, this protein was expressed in paired *Xenopus* oocytes where it forms gap junction channels (Phelan et al., 1998a). In 1993, a gene called *unc-7* was identified in *Caenorhabditis elegans* (*C. elegans*), which was required for coordinated locomotion (Starich et al., 1993). With this background, the new family of genes was first proposed as OPUS for *O*gre (gene that encodes the innexin 1 protein), *P*as, *unc-7* and *S*haking-B, but subsequently, the name was changed to innexins for *in*vertebrate homologs of con*nexins* (Phelan et al., 1998b). Notably, studies previous to the description of the innexins reported the existence of gap junctions in invertebrates without being associated with any specific function, such as the gap junctions in seminiferous epithelium of *Triatoma infestans* (Miranda and Cavicchia, 1988), intestinal tissue of *Sagitta setosa* (Duvert et al., 1980), photoreceptor cells of *Apis mellifera* (Pabst and Kral, 1989), giant axon and inner nerve of *Aglanta digitale* (Weber et al., 1982), endoderm of *Polyorchis penicillatus* (King and Spencer, 1979), and ciliated cells of *Pleurobrachia bachei* (Satterlie and Case, 1978). We currently know many functions of the innexins as cell-cell communication pathways mediated by gap junction channels and as well as hemichannels.

## Hemichannels

A hemichannels correponds to one half of a gap junction channel, and plays a important role as autocrine/paracrine cellular communication pathways (Sáez et al., 2003). Hemichannels are permeable to ions and several metabolic and signaling molecules such as glucose, glutamate, glutathione, adenosine, NAD^+^ and ATP among others (Sáez et al., 2003). Structurally, hemichannels are formed by oligomers of connexin or innexin proteins (Sáez et al., 2003). These proteins may co-oligomerize into the same (homomeric) or mixed (heteromeric) hemichannels (Falk et al., 1997). In invertebrates, hemichannels are formed exclusively by innexin proteins (Phelan et al., 1998b). Although they bear no sequence homology with connexins, these genes encode proteins that compose hemichannels in insects and other prechordates (Phelan et al., 1998b).

## Innexin genes and expression

Innexin genes have been found in the phyla *Arthropoda* (Ganfornina et al., 1999; Stebbings et al., 2002; Hong et al., 2008; Calkins et al., 2015), *Nematoda* (Starich et al., 2001), *Chordata* (White et al., 2004), *Annelida* (Potenza et al., 2003; Kandarian et al., 2012), *Platyhelminthes* (Zurabian et al., 2008), *Cnidaria* (Takaku et al., 2014), and *Mollusca* (Kelmanson et al., 2002). Eight genes have been identified in *Drosophila melanogaster* (*D. melanogaster*) (Stebbings et al., 2002), 25 in *C. elegans* (Starich et al., 2001) and 21 in *Hirudo verdana* (*H. verdana*) (Kandarian et al., 2012). In the phylum *Platyhelminthes*, the innexin genes have been identified in *Girardia tigrina, Dugesia japonica* and *Schmidtea mediterranea* (Panchina et al., 2000; Nogi and Levin, 2005; Oviedo and Levin, 2007). Important to mention is that formats of gene and protein names of innexin depends on the species. For example, in worms a gene name is a three or four letter code and a number with a hyphen between them, all italicized, whereas a protein name uses capital letters and is not italicized (Horvitz et al., 1979). In fly, a gene name is a three letter code and a number, whereas a protein name uses the first capital letter plus a number (Ashburner et al., 2000). The formats for the other species did not follow the formats described above. In *Hirudo medicinalis* (*H. medicinalis*), analyses of gene structure showed that depending on the leech innexin gene, they can contain from 0 to 6 introns (Kandarian et al., 2012). For example, *Hm-inx14, Hm-inx15, Hm-inx16*, and *Hm-inx17* contain 1 exon, whereas *Hm-inx3, Hm-inx7, Hm-inx9a, Hm-inx9b*, and *Hm-inx13* contain 7 exons, suggesting that number of introns does not correlate with protein size (Kandarian et al., 2012). In *D. melanogaster*, innexin genes are located on the X chromosome in two small clusters, with three genes sitting within a 10 kilobase distance from one another (Curtin et al., 1999). The authors suggest an evolutionary origin of these genes via local duplication (Curtin et al., 1999). In *Bombyx mori* (*B. mori*), the *BmINX2* gene has one exon; whereas the *BmINX4* gene has four exons and three introns (Hong et al., 2008). In *H. verdana, in situ* hybridization showed that ~50% of the expressed innexins are detectable in multiple tissues (Kandarian et al., 2012). Innexins are expressed in the central nervous system of *H. medicinalis* (Dykes et al., 2004). Using DIG-labeled antisense RNA probes, Dykes et al. (2004) showed that *Hm-inx1* is expressed in neurons but not in glial cells. By contrast, *Hm-inx2* is expressed in glia but not in neurons (Dykes et al., 2004). In *D. melanogaster, in situ* hybridization studies showed that pas-related proteins (prp) 7 and 33 are expressed in the central nervous system, gut and epidermis (Curtin et al., 1999). Notably, each innexin gene has a different pattern of expression in each of the tissues (Curtin et al., 1999). In *B. mori, in situ* hybridization analysis revealed that *BmINX2* and *BmINX3* are highly expressed in the nervous system during embryogenesis (Hong et al., 2008). *BmINX4* is transiently expressed at the germ-band formation stage and *BmINX3* is restricted to the blastokinesis stage (Hong et al., 2008). Moreover, after fifth instar larvae, *BmINX2, BmINX3*, and *BmINX4* are expressed in ovary and testis (Hong et al., 2009). BmInx2 or BmInx3 fused to green fluorescent protein (GFP) showed a cytoplasmic localization, whereas the BmInx4-GFP showed a plasma membrane localization specifically at the contact site of the cells (Hong et al., 2009). Unfortunately, the authors did not describe whether GFP fusion proteins are functional and reflect subcellular localization patterns of native proteins. However, in previous studies, they describe that during the development of the ovarian chamber, BmInx2 is highly expressed at the interface between the oviduct and cytoplasm, suggesting that BmInx2 could be normally located in the plasma membrane (Hong et al., 2008). In *C. elegans*, expression of GFP under the control of individual innexin promoters showed that innexins are found in all cell types and tissues of the worm with dynamic expression patterns (Altun et al., 2009). Interestingly, innexin are expressed in cell types that have not been described as forming intercellular channels, for example migratory distal tip cells or sperms (Altun et al., 2009). In the phylum *Platyhelminthes*, innexins are expressed in the nervous system, intestine, and mesenchymal tissue (Panchina et al., 2000; Nogi and Levin, 2005; Oviedo and Levin, 2007). Electron microscopy images showed gap junctions in immature proglottid and in the neck of *Taenia solium*. However, their functions have not been elucidated (Zurabian et al., 2008).

## Topology and regulation of innexons

The membrane topology of innexin proteins is similar to that of connexins or pannexins, with 4 transmembrane domains, 2 extracellular loops, and intracellular C and N-terminal domains (Barnes, 1994; Panchina et al., 2000; Bruzzone et al., 2003; Beyer and Berthoud, 2018). These proteins present highly conserved residues among which are two well-conserved pairs of cysteyl residues in extracellular loop (EL) 1 and EL2, a proline residue in the transmembrane domain (TMD) 2, and a tryptophan residue in the TMD 4 (Yen and Saier, 2007). The proteins also show a highly conserved YYQWV pentapeptide located in the TMD2, which is called “innexin motif” (Phelan, 2005). Structural characterization of channels formed by *C. elegans* INX-6 showed the structure of the intercellular channel consists of 16 subunits formed by two innexons of 8 subunits each (Oshima, 2017). Recently, this structure was confirmed by cryo-electron microscopy studies (Oshima, 2017). The hexadecameric structure of the INX-6 gap junction channel appears to be similar to that of connexin 26 (Cx26) formed gap junction channel expressed in vertebrates (Maeda et al., 2009; Bennett et al., 2016; Oshima, 2017). For example, both Cx26 and INX-6 have a highly similar monomeric structural organization (Oshima, 2017). Also, the special distribution of the two disulphide bonds formed by the cysteines of EL1 and EL2 in INX-6 well corresponds to two of the three disulphide bonds formed by the cysteines of EL1 and EL2 in Cx26 (Oshima, 2017). Interestingly, the mutation of proline residue located in the TMD2 causes a gap junction protein with dominant negative properties (Yazdani et al., 2013). In 2011, Depriest and collaborators performed tryptophan scanning mutagenesis assays to determine the relationship between structure and function of *D. melanogaster* innexin Shaking-B (Lethal) (Depriest et al., 2011). These studies allowed to identify that tryptophan substitution at several sites in TMD1 (H27, T31, L35, or S39) alter channel properties (Depriest et al., 2011). Pharmacological studies indicate that innexons are sensitive to carbenoxolone in a concentration-dependent way (Bao et al., 2007; Luo and Turnbull, 2011) and to Brilliant Blue G (Bao et al., 2007; Samuels et al., 2013; Dahl and Muller, 2014). In oocytes injected with *Hm-inx2* from *H. medicinalis* exposed to a solution with high K^+^ generated an output of ATP (Bao et al., 2007). In relation to electrophysiological properties, innexons from *H. medicinalis* exhibit multiple subconductance states with maximal single channel conductance of 500 pS for *Hm-inx2, Hm-inx3*, or *Hm-inx6* and ~250 pS for *Hm-inx1* (Bao et al., 2007). With regard to the regulatory mechanisms of innexons, they open in response to mechanical stress, increased [K^+^]_o_, membrane depolarization (+20 mV or higher), and increased cytoplasmic Ca^2+^ concentrations (Bao et al., 2007; Dahl et al., 2013, Dahl and Muller, 2014). The amino terminus domain participates in the regulation of voltage gating and junctional rectification of Shaking B (Marks and Skerrett, 2014). By contrast, the channel function is attenuated by arachidonic acid (Samuels et al., 2013), lipopolysaccharide (Luo and Turnbull, 2011) and cytoplasmic acidification (Bao et al., 2007; Dahl and Muller, 2014). Additionally, aptamers of anti-innexin 2 specifically inhibit the interaction of the Inx2 and Inx3 carboxyl-termini (Knieps et al., 2007).

## Innexins in embryogenesis

The role of innexins in embryogenesis is well described in the *D. melanogaster* model (Lipshitz and Kankel, 1985; Ostrowski et al., 2008; Giuliani et al., 2013). The expression of Inx1, Inx2, Inx3, and Inx7 in the ectoderm has been described (Ostrowski et al., 2008, Giuliani et al., 2013). In 1985, mutant studies showed that individuals with a mutation in the *org* gene, which codes for an innexin, had serious defects in the development of the central nervous system (Lipshitz and Kankel, 1985). The mutation affects the assembly of the adult optic lobes during the larval period (Lipshitz and Kankel, 1985). Additionally, mutations of Inx3 result in embryos with dorsal closure defects or embryos producing cuticles with strong head involution (Giuliani et al., 2013). These defects were rescued with overexpression of Inx3 (Giuliani et al., 2013). Moreover, a down-regulation of Inx7 expression causes a severe disruption of embryonic development of the nervous system (Ostrowski et al., 2008). Additionally, down-regulation of Inx2 expression in glial cells causes a significant reduction in the size of the nervous system of the larval stage (Holcroft et al., 2013). Studies of loss and gain in function showed that depleting Inx2 or Inx3 reduces the eye size, whereas elevating Inx2 or Inx3 level increases the eye size (Richard and Hoch, 2015; Richard et al., 2017). Innexins are also important for polarity and organization of the embryonic epidermis in *Drosophila* (Bauer et al., 2004; Lehmann et al., 2006). For example, down-regulation of Inx2 or Inx3 causes severe developmental defects in epithelial morphogenesis, and mutants have a large hole in the cuticle or even a complete loss of cuticle (Bauer et al., 2004; Lehmann et al., 2006). All these defects were rescued when one paternal copy of Inx2 was added back to the maternal null background (Bauer et al., 2004). Moreover, Inx2 mutants exhibit a feeding defect (Bauer et al., 2002). This alteration is caused by the inability to migrate and invaginate the epithelial cells during proventriculus organogenesis (Bauer et al., 2002). Later, Inx2 was discovered to be essential for transcriptional activation of hedgehog, wingless, and delta pathways during foregut morphogenesis (Lechner et al., 2007). Innexin proteins are relevant for intercellular transport of nucleotide sugars in the epithelium of the wing during development of *D. melanogaster* (Ayukawa et al., 2012). Down-regulation of Inx2 expression results in loss of wing-margin tissue caused by interruption of the notch signaling pathway (Ayukawa et al., 2012). The authors suggested that intercellular supply of GDP-L-fucose via innexin-formed gap junction is required for the O-fucosylation of Notch (Ayukawa et al., 2012). The participation of innexin in early development has also been demonstrated in *C. elegans*. For example, mutant worms of INX-3 exhibit defects during embryonic morphogenesis such as the failure of the mid-body to elongate properly or the failure of the pharynx to attach to the anterior, causing the death of most mutants (Starich et al., 2003). Additionally, innexin coordinate left-right neuronal asymmetry in the developing nervous system for which the innexin NSY-5 is required for AWC (amphid wing “C”) olfactory neurons to establish asymmetric patterns of gene expression during embryogenesis (Chuang et al., 2007). In the red flour beetle *Tribolium castaneum*, an innexin orthologous named TC011061 is necessary for embryogenesis (van der Zee et al., 2015). Down-regulation of expression of TC011061 causes instability of the blastoderm resulting in defect in the cellularization process (van der Zee et al., 2015).

## Innexins in the reproduction system

Several studies demonstrate the participation of innexins in the reproduction system (Tazuke et al., 2002; Edmonds et al., 2011; Magnusson et al., 2011; Starich et al., 2014; Gabrieli et al., 2016). In *D. melanogaster*, Inx4 mutation causes tiny gonads and sterility (Tazuke et al., 2002). Similar results were obtained in the malaria vector *Anopheles gambiae* (*A. gambiae*) in which silencing AGAP006241 (a putative innexin orthologue) causes a phenotype characterized by a defect in gonad development; specifically, males do not present spermatogenesis, and females do not present follicles (Magnusson et al., 2011). Moreover, in the Mediterranean fruit fly *Ceratitis capitata*, down-regulation of Inx5 expression results in males without sperm and females lack mature eggs (Gabrieli et al., 2016). In *C. elegans*, innexins are relevant for proliferation of germ line stem cells and gametogenesis (Starich et al., 2014). Gap junctions composed of INX-8 and INX-9 in the soma or INX-14 and INX-21 in the germ line paticipate in the differentiation and proliferation of germ line stem cells, whereas gap junctions composed of somatic INX-8 and INX-9 or germ line INX-14 and INX-22 participate in the negative regulation of oocyte meiotic maturation (Whitten and Miller, 2007; Starich et al., 2014). Additionally, INX-14 promotes sperm guidance in the reproductive tract of *C. elegans* (Edmonds et al., 2011). In *D. melanogaster*, Inx2 is expressed in follicle cells during oogenesis (Stebbings et al., 2002). Moreover, immuneutralization assays using antibodies against Inx2 showed a reduction of cell-cell transfer of Lucifer yellow between oocyte and follicle cells and consequently defects in oocyte growth (Bohrmann and Zimmermann, 2008).

## Innexins in the nervous system

### Synaptogenesis

In *C. elegans*, innexin UNC-7 and UNC-9 are required for presynaptic differentiation during synaptogenesis (Yeh et al., 2009). Additionally, innexins UNC-7 and UNC-9 regulate the distribution and size of active zones at neuromuscular junctions (Yeh et al., 2009). Down-regulation of *Sg*-INX1, *Sg*-INX2, *Sg*-INX3, or *Sg*-INX4 expression inhibits synaptogenesis in locust neural cultures of *Schistocerca gregaria* (Anava et al., 2013). In *D. melanogaster*, innexins are expressed in pre- and post-synaptic neurons and are relevant for rectifying electrical synapses in giant fiber system (Phelan et al., 2008). In fact, the amount and localization of presynaptic gap junctions are regulated by netrin and frazzled in the giant fiber system (Orr et al., 2014). According to the authors, frazzled or netrin loss-of-function mutants exhibit an altered distribution of the gap junction in the giant fiber system (Orr et al., 2014). In the leech *H. medicinalis*, down-regulation of *Hm-Inx1* expression in retzius neurons decreases their electrical coupling and formation of chemical synapses (Todd et al., 2010).

### Synaptic transmission

Innexins are important in synaptic transmission. For instance, in *C. elegans*, gap junctions serve as an amplifier of chemical transmission between premotor interneurons (AVA) and downstream motor neurons (A-MNs; Liu et al., 2017). Interestingly, gap junctions between AVA and A-MNs only allow antidromic current and disrupting electrical coupling inhibits chemical transmission. In contrast, disrupting chemical synapses has no effect on the electrical coupling Liu et al., 2017. In *D.melanogaster, two* different isoforms of the innexin Shaking-B have been described to form rectifying electrical synapses in giant fiber system (Phelan et al., 2008). A Shaking-B (Neural+16) isoform was shown to be required presynaptically, while Shaking-B (Lethal) isoform is required postsynaptically (Phelan et al., 2008). When both proteins are expressed *in vitro* in neighboring cells, they form heterotypic gap junction channels that are asymmetrically gated by voltage and exhibit classical rectification (Phelan et al., 2008).

### Rhythmic central pattern generator networks

In crustaceans, innexins are important in rhythm-generating networks (Shruti et al., 2014; Otopalik et al., 2017). For example, Inx1, Inx2, and Inx3 are expressed in stomatogastric ganglion of *Cancer borealis* (*C. borealis*) (Otopalik et al., 2017). Also, electrophysiological studies have demonstrated gap junctional coupling between stomatogastric ganglion neurons (Shruti et al., 2014). The stomatogastric ganglion are responsible for gastric movements in crustaceans (Maynard and Dando, 1974; Marder and Bucher, 2007; Shruti et al., 2014; Otopalik et al., 2017). Additionally, Inx1, Inx2, and Inx3 expression is found in large motor neurons of the cardiac ganglion of *C. borealis* (Otopalik et al., 2017). Moreover, cardiac ganglion is composed of 6–16 neurons that autonomously provide rhythmic action potentials to activate the heart muscle (Otopalik et al., 2017). In the desert locust *S. gregaria*, innexins are important in a frontal ganglion and central pattern-generating networks (Anava et al., 2009). Functional studies showed that innexins allow functional electrical coupling between neurons in a frontal ganglion that regulates two fundamental behaviors such as feeding and molting (Anava et al., 2009).

### Behavior

In *C. elegans*, gap junctions formed by UNC-7 or UNC-9 are required for coordinated behaviors (Chen et al., 2007; Kawano et al., 2011; Jang et al., 2017). For example, down-regulation of UNC-9 expression results in locomotion defects and loss of aggregation of worms (Jang et al., 2017). Moreover, *unc-7* or *unc-9* mutants or double-null mutants show greatly reduced forward movement, characterized by a movement defect described as kinking (Starich et al., 1993; Kawano et al., 2011). Additionally, mutations of UNC-1, a stomatin-like protein required for the function of UNC-9 gap junctions, inhibit locomotion (Chen et al., 2007). Immunohistochemistry studies show that UNC-1 and UNC-9 colocalized at intercellular junctions in neurons and body wall muscle cells (Chen et al., 2007). Experiments on the cnidarian *Hydra polyps* (*H. polyps*) demonstrate that innexin-formed gap junctions are essential for coordinated behaviors (Takaku et al., 2014). For example, treatment of live animals with Inx2 antibody reduces spontaneous body column contractions in *H. polyps* (Takaku et al., 2014).

### Memory

In *D. melanogaster*, down-regulation of Inx7 in the anterior paired lateral neurons and knockdown of Unc6 in the dorsal paired medial neurons cause the flies to fail to form anesthesia-sensitive memory (Wu et al., 2011). The authors showed that heterotypic gap junctions between the dorsal paired medial and anterior paired lateral neurons are relevant for memory formation in *D. melanogaster* (Wu et al., 2011).

### Auditory sensory

In *D. melanogaster*, down-regulation of several innexins affects the escape response to sound (Pézier et al., 2016). For example, down-regulation of Ogre, Inx3, or Inx6 expression causes a reduction in the amplitude of the action potential recorded in response to sound (Pézier et al., 2016). Because innexin proteins permit the synaptic transmission between Johnston's Organ neurons and giant fiber, their absence affects the escape response to sound (Pézier et al., 2016).

## Innexins in the muscle system

In insects, the striated muscle cells are connected by gap junctions formed by innexins (Yoshimura et al., 2017). This was demonstrate by microinjection of Lucifer yellow into the muscle cells in *Periplaneta americana* (Yoshimura et al., 2017). In *C. elegans*, electrophysiological studies show that muscle cells are electrically coupled through gap junctions formed by innexins (Liu et al., 2006, 2013b). Interstingly, the specific loss of UNC-9 reduces locomotion velocity of worms (Liu et al., 2006). Based on mutant studies, a total of six innexins contribute to the coupling of ventral body-wall muscle cells of *C. elegans* (Liu et al., 2013b). The innexins identified were UNC-9, INX-1, INX-10, INX-11, INX-16, and INX-18 (Liu et al., 2013b). Additionally, gap junctions mediate the synapsis between pharyngeal motor neuron M4 and pharyngeal terminal bulb muscles (Steciuk et al., 2014). An *eat-5* mutant worm exhibits few pharyngeal terminal bulb contractions and is unable to grow well (Steciuk et al., 2014). In inx-6 mutant, electrical coupling decreases between the anterior pharyngeal muscles, causing a premature relaxation in the anterior pharynx and unsynchronized pharyngeal muscle contraction, interfering with feeding (Li et al., 2003).

## Innexins in the renal system

Innexins are relevant for electrical coupling in the renal tubules of mosquitoes (Loewenstein et al., 1965; Weng et al., 2008). Moreover, renal tubules of *Aedes aegypti* (*A. aegypti*) express transcripts for *AeInx1, AeInx2, AeInx3*, and *AeInx7* genes (Weng et al., 2008). Additionally, a gap junction blocker results in inhibition of diuresis in mosquitoes, which demonstrates the participation of this intercellular coupling based on innexins in the regulation of metabolism in renal tubules (Piermarini and Calkins, 2014). In *in vivo* assays, the injection of *A. aegypti* with carbenoxolone significantly reduced the diuresis (Piermarini and Calkins, 2014).

## Innexin in the digestive system

In *A. aegypti*, innexin participate in contractile properties of the ventral diverticulum (Calkins et al., 2017). The ventral diverticulum is the primary storage organ for imbibed sugar in the midgut for digestion, and the addition of carbenoxolone reduces the ventral diverticulum contraction rates (Calkins et al., 2017). Moreover, preincubation with carbenoxolone prevents the increase in contraction rates of the ventral diverticulum in response to serotonin (Calkins et al., 2017). Innexins also participate in the mechanism of defecation in worms (Peters et al., 2007). Mutants for *inx-16* show a constipated phenotype and exhibit multiple defects in the defecation cycle (Peters et al., 2007). Studies carried out with GFP under the *inx-16* promoter demonstrated that INX-16 is located in the intestine of worms, specifically in the cell-cell contact zone (Peters et al., 2007). Additionally, *inx-16* mutants present an altered spatial and temporal pattern of calcium waves along the intestine (Peters et al., 2007). Because the defecation mechanism is coordinated by the propagation of intercellular calcium waves in the intestine, these results suggest that innexins are relevant in this process (Teramoto and Iwasaki, 2006; Peters et al., 2007).

## Innexin in the immune system

Studies performed in *Scylla paramamosain* (*S. paramamosain*) have shown that channels formed by innexin are critical to the immune response (Wang et al., 2015). High levels of mRNA for Sp-inx2 were identified in hemocytes, which are major invertebrate innate immune cells (Wang et al., 2015). Additionally, Sp-inx2 was up regulated in hepatopancreas tissue, gill and hemocyte with the challenge of either *Vibrio alginolyticus* or *Vibrio parahaemolyticus*, suggesting their participation in the immune response of *S. paramamosain* against bacterial agents (Wang et al., 2015). According to the authors, lipopolysaccharides increase the levels of mRNA transcripts and protein of Sp-inx2 in hemocytes (Wang et al., 2015). Notably, in HeLa cells transfected with Sp-inx2, a bacterial lipopolysaccharide reduces the activity of Sp-inx2-formed hemichannels (Wang et al., 2015). Additionally, an immune challenge with the parasitoid *Microplitis bicoloratus* produces down-regulation of Sl-inx1, Sl-inx2, and Sl-inx3 expression in hemocytes of *S. litura* (Li et al., 2014; Pang et al., 2015). Innexins also participate in the process of apoptosis in hemocytes (Liu et al., 2013a; Wang et al., 2015). For example, the ectopic expression of Sl-inx2 or Sl-inx3 of hemocytes of *S. litura* promotes apoptosis in the insect cell line Spli221 (Liu et al., 2013a). Additionally, the ectopic expression of Sp-inx2 of *S. paramamosain* promotes apoptosis in HeLa cells and epithelioma papulosum cyprinid cell lines (Wang et al., 2015).

## Innexins in regeneration

The participation of innexins during the regeneration of the planarian *D. japonica* has been described (Oviedo et al., 2010). The exposure to octanol, a blocker of gap junctions, after transversal amputation results in alteration of anterior/posterior polarity during regeneration, which generates ectopic anterior blastemas at posterior-facing wounds that develops into head of *D. japonica* (Oviedo et al., 2010). Moreover, down-regulation of *Dj-Inx-5, Dj-Inx-12*, or *Dj-Inx-13* expression causes individuals with an inverted anterior-posterior axis and bipolar head regeneration (Oviedo et al., 2010). Additionally, exposure to heptanol, a blocker of gap junctions, results in an anteriorization of both blastemas, generating a loss of tail development or appearance of an ectopic eye, pharynx, and complete head at the posterior in *D. japonica* (Nogi and Levin, 2005).

## Innexins a therapeutic targets of parasitic diseases

### Malaria disease

Clinical studies find that probenecid, a blocker of hemichannels formed by innexins, has a powerful antiparasitic effect (Nzila et al., 2003; Sowunmi et al., 2004; Masseno et al., 2009). For example, probenecid increases the sensitivity of a highly resistant plasmodium strain against antifolate components (Nzila et al., 2003). The mechanism has not been elucidated, but it is not associated with sensitivity status of the parasite or with alterations of the dihydrofolate reductase or dihydropteroate synthase (Nzila and Al-Zahrani, 2013). In fact, it has been suggested that it is a transport-based mechanism linked to folate salvage (Nzila and Al-Zahrani, 2013). In clarifying whether probenecid affects the activity of gap junction channels, hemichannels or both, it has been reported that probenecid inhibits the activity of innexin- or pannexin-formed hemichannels (Wang et al., 2015). Moreover, the effect of probenecid on innexin-formed gap junction channel has not been evaluated. Also, it has been described that Cx46 or chimera Cx32E143-formed hemichannels are not affected by probenecid (Silverman et al., 2008). Moreover, in human erythrocytes, antimalarial drugs such as artemisinin and artesunate reduce the activity of channels formed by pannexin-1, a homolog of innexin in vertebrates (Dahl et al., 2013).

### Chagas disease

Suramin, a blocker of hemichannels, has a powerful trypanocidal effect (Bisaggio et al., 2006). For example, the exposure of *T. cruzi* infected-LLC-MK2 cells to suramin causes a partial or complete detachment of the flagellum from the cell body in trypomastigote forms (Bisaggio et al., 2006). Although the mechanism has not been elucidated, it has been described that suramin affects the activity of several enzymes such as kinases, phosphatases, ATPases, oxidases and phospholipases (Voogd et al., 1993). Furthermore, suramin acts as antagonist of P2X and P2Y purinoceptors (Voogd et al., 1993). In trypanosomes, a prolonged incubation (5–7 days) with suramin causes an increase in Mg^2+^-dependent ecto-ATPase activity (Bisaggio et al., 2003). Although the effect of suramin on the innexin-formed hemichannels has not been described, it has been described that suramin blocks the permeability of Cx43 hemichannels activated by removal of extracellular Ca^2+^ without much effect on gap junctional communication (Chi et al., 2014). Currently, suramin is used for treatment of parasitic diseases caused by protozoa (Sowunmi et al., 2004).

### Arthropod vectors

Pharmacological inhibitors of gap junctions are potential insecticides (Calkins and Piermarini, 2015). For example, mefloquine and meclofenamic acids are toxic to adult female *A. aegypti* and upon topically application to the cuticle, carbenoxolone showed full efficacy (Calkins and Piermarini, 2015). The authors suggest that the mechanism would be an alteration of renal function in the mosquito (Calkins and Piermarini, 2015).

## Concluding remarks

The innexin proteins are members of the gap junction family found in invertebrates and are involved in a series of biological functions. Many studies show the importance of the formation of innexin gap junctions between neighbor cells, demonstrating the necessity of intercellular communication to coordinate different processes, such as embryonic development, which emphasizes their role in morphogenesis and neurogenesis. Additionally, gap junctions within the adult stage participate in physiological functions, behavior, and memory. Fewer reports describe the importance of the no-junctional channels formed by innexin proteins, although specifically in processes of immune response and apoptosis.

## Author contributions

JG, JCS, and JLV contributed conception and design of the study. JG and JLV organized the database. JG wrote the first draft of the manuscript. IB, JCS, and JLV wrote sections of the manuscript. All authors contributed to manuscript revision, read, and approved the submitted version.

### Conflict of interest statement

The authors declare that the research was conducted in the absence of any commercial or financial relationships that could be construed as a potential conflict of interest.
